# Convenient methods for preparing π-conjugated linkers as building blocks for modular chemistry

**DOI:** 10.3762/bjoc.5.11

**Published:** 2009-04-14

**Authors:** Jiří Kulhánek, Filip Bureš, Miroslav Ludwig

**Affiliations:** 1 Institute of Organic Chemistry and Technology, Faculty of Chemical Technology, University of Pardubice, nám Čs. legií 565, Pardubice, 532 10, Czech Republic

**Keywords:** boronic acid, donor/acceptor, linker, Sonogashira reaction, property tuning, push-pull, Suzuki–Miyaura reaction

## Abstract

Simple, straightforward and optimized procedures for preparing extended π-conjugated linkers are described. Either unsubstituted or 4-donor substituted π-linkers bearing a styryl, biphenyl, phenylethenylphenyl, and phenylethynylphenyl π-conjugated backbone are functionalized with boronic pinacol esters as well as with terminal acetylene moieties allowing their further use as building blocks in Suzuki–Miyaura or Sonogashira coupling reactions.

## Introduction

**Figure 1:**
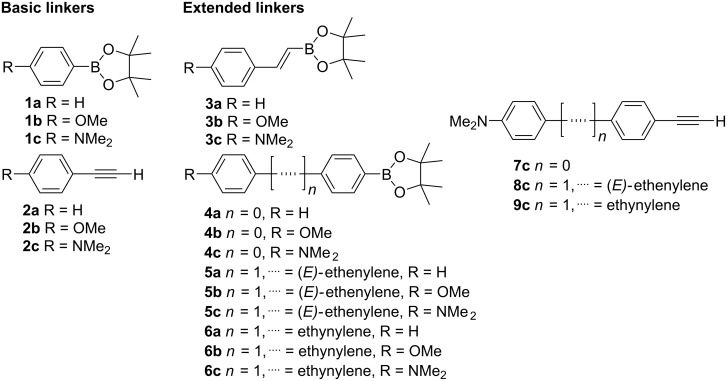
Basic and newly proposed π-conjugated linkers designed for the Suzuki–Miyaura and Sonogashira cross-coupling.

Development of new organic compounds with improved and advanced properties is one of the most important goals of modern material chemistry. Organic chemists steadily attempt to design and synthesize novel and well-defined organic push-pull systems with prospective applications as chromophores for nonlinear optics (NLO) [1–5], dyes [6], electronic and photonic devices [7–8], organic light-emitting diodes (OLED) [9] or functional polymers [10–13]. A typical push-pull chromophore consists of a polar A-π-D system with a planar π-system end-capped by a strong electron donor (D) and a strong electron acceptor (A). The π-conjugated system ensuring charge-transfer (CT) between the donor (D = NR_2_, OR groups etc.) and the acceptor (A = NO_2_, CN groups etc.) is most commonly comprised of double and triple bonds, aromatic and heteroaromatic rings as well as their combinations [14–19]. A typical synthetic approach to CT chromophores involves either a stepwise formation of the target molecule [19–20] or a separate preparation of the donor as well as the acceptor moieties and their final combination [21–22]. It is already well known that the HOMO/LUMO gap and polarizability of the push-pull systems with the given donors and acceptors can be finely tailored by the extension or shortening of the π-conjugated path between the donor and acceptor [19,21,23–25]. Thus, the latter modular synthetic approach seems to be more suitable for the property tuning described above. The final combination, C–C bond formation, of the donor and acceptor chromophore moieties is usually accomplished by cross-coupling reactions, in particular by the Suzuki–Miyaura [26–27] or the Sonogashira [28] reactions. Consequently, the availability of the suitably substituted π-conjugated linkers of various lengths bearing boronic ester functionality or terminal acetylene is crucial for such a synthetic approach. Hence, we report here a convenient synthesis as well as characterization of either unsubstituted (R = H) or donor substituted (R = NMe_2_, OMe) π-conjugated linkers designed for the Suzuki–Miyaura and Sonogashira cross-couplings with a systematically varied and enlarged π-conjugated path (Figure 1).

Whereas the simplest linkers such as 4-substituted phenylboronic pinacol esters **1a**–**c** [29] and ethynylbenzenes **2a**–**c** [30] are well known and also commercially available, the proposed dioxaborolanes **3**–**6** feature styryl (series **3**), biphenylyl (series **4**), (*E*)-phenylethenylphenyl (series **5**), and phenylethynylphenyl (series **6**) linkers and H (series **a**), OMe (series **b**), and NMe_2_ (series **c**) groups as the substituent R, respectively (Figure 1, Table 1). The terminal acetylenes **7c**–**9c** possess only the strongest NMe_2_ donor and have an identical backbone to the one described above (Figure 1, Table 1).

**Table 1:** Optimized synthetic procedures and yields for the preparation of **3**–**9**.

Entry	Product (R)	Method	Yield (%)

1	**3c** (NMe_2_)	A	73^a^
2	**4b** (OMe)	B	81^a^
3	**4c** (NMe_2_)	B	83^a^
4	**5a** (H)	C	76^b^
5	**5b** (OMe)	C	69^a^
6	**5c** (NMe_2_)	C	82^a^
7	**6a** (H)	D	78^a^
8	**6b** (OMe)	D	72^a^
9	**6c** (NMe_2_)	D	91^a^
10	**10**/**7c**	E	98/92^c^
11	**11**/**8c**	E	97/91^c^
12	**12**/**9c**	E	99/89^c^

^a^Yield of the final coupling step. ^b^Horner–Wadsworth–Emmons reaction. ^c^Yield of the Sonogashira cross-coupling (**10**–**12**) and final TMS-group removal to the terminal acetylenes **7c**–**9c**.

## Results and Discussion

### Synthesis of boronic pinacol esters **3**–**6**

**Scheme 1:**
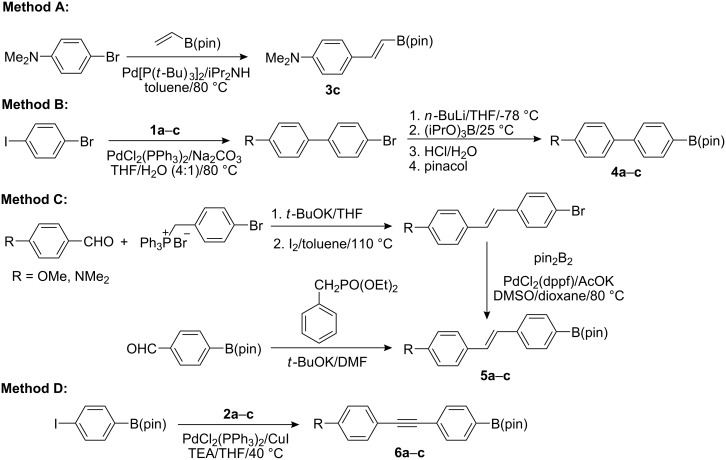
Convenient synthetic methods leading to π-linkers **3**–**6**.

Whilst two styryl dioxaborolanes **3a**–**b** are known [31–32] and commercially available, the *N*,*N*-dimethylamino substituted derivative **3c** needed to be synthesized. In order to achieve pure (*E*)-**3c**, at first, a hydroboration of the commercially available terminal acetylene **2c** with catecholborane was examined. Despite all attempts to optimize the reaction conditions, **3c** could not be prepared this way and was not even detected in the crude reaction mixture. Thus the above hydroboration reported by Perner and co-workers [33] proved to be infeasible. However, in light of the report by Itami and Yoshida [34], we attempted the Mizoroki–Heck C–H arylation of 4-bromo-*N*,*N*-dimethylaniline with an equimolar amount of vinylboronate pinacol ester leading to the desired **3c** in 73% yield (Scheme 1, Method A).

4-Substituted 4′-bromobiphenyl intermediates necessary for the preparation of **4a**–**c** were synthesized by the Suzuki–Miyaura cross-coupling of 1-bromo-4-iodobenzene with the corresponding boronic acids/esters **1a**–**c** in the yields of 82, 84, and 91%, respectively. A routine procedure involving a lithiation and reaction with triisopropyl borate followed by esterification with pinacol afforded dioxaborolanes **4b**–**c** in the yields of 81 and 83% (Scheme 1, Method B). The biphenyl-4-boronic acid corresponding to **4a** was also commercially available while **4b** was reported as a side product [35] without full characterization.

Whereas **5a** was easily accessible as a pure (*E*)-product from the pinacol ester of 4-formylphenylboronic acid and diethyl benzylphosphonate through the Horner–Wadsworth–Emmons reaction in 76% yield [36], methoxy and *N*,*N*-dimethylamino substituted (*E*)-4-bromostilbenes were synthesized from the corresponding benzaldehydes and 4-bromobenzyl(triphenyl)phosphonium bromide [37] by the Wittig reaction [37–38] in 37 and 54% yields, respectively. In contrast to the Horner–Wadsworth–Emmons reaction, this procedure afforded both (*E*)- and (*Z*)-stilbenes that were isomerized by heating with traces of iodine in toluene to afford pure (*E*)-stilbenes [38]. However, compared to *N*,*N*-dimethylaminostilbene, the isomerization of 4-bromo-4′-methoxystilbene required twelve times the prolonged reaction time (4 vs. 48 h) while the isomerisation of unsubstituted 4-bromostilbene did not take a place at all. Hence, **5a** had to be prepared by the Horner–Wadsworth–Emmons reaction described above. These substituted 4-bromostilbenes could be most effectively converted to target pinacol esters **5a**–**c** via borylation (Scheme 1, Method C) utilizing bis(pinacolato)diboron (pin_2_B_2_) [39] in a mixed solvent system DMSO/dioxane ensuring good solubility. It should be noted here that **5a**–**c** were also accessible via the routine sequence showed for Method B. On the contrary, borylation of 4-bromobiphenyls with pin_2_B_2_ (Method C) yielded only traces of **4a**–**c**.

Linear phenylethynylphenyl π-linkers **6a**–**c** were gained by a Sonogashira cross-coupling between the pinacol ester of 4-iodophenylboronic acid [39] and terminal acetylenes **2a**–**c** (Scheme 1, Method D) in 72–91% yield. Table 1 (entries 1–9) summarizes the used synthetic methods and yields for the particular dioxaborolanes **3**–**6**.

### Synthesis of terminal acetylenes **7**–**9**

**Scheme 2:**
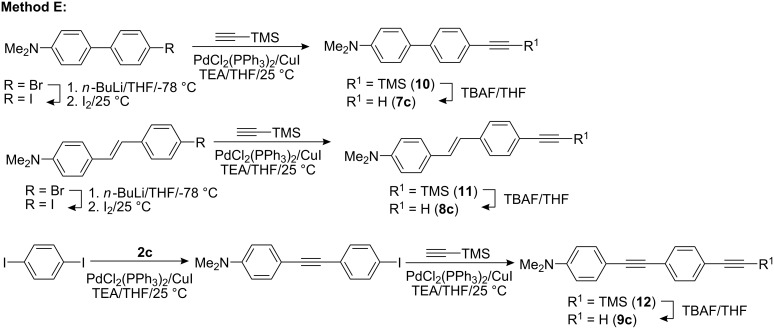
Sonogashira cross-coupling leading to π-linkers **7c**–**9c**.

Synthesis of *N*,*N*-dimethylamino substituted terminal acetylenes **7c**–**9c** was accomplished by Sonogashira cross-coupling as shown on the Scheme 2 (Method E). The reaction utilizes the 4-bromo derivatives used as precursors for the construction of dioxaborolanes **4**–**6** and the product of the Sonogashira cross-coupling between **2c** and 1,4-diiodobenzene (54% yield). Since the Sonogashira reaction between bromo derivatives and trimethylsilylacetylene proved to be sluggish and low yielding (even with a large excess of acetylene and elevated temperature), the bromo derivatives were converted to the corresponding iodo derivatives by lithiation and quenched with iodine (see Supporting Information File 1). Thus the Sonogashira reaction on iodo derivatives smoothly furnished TMS-protected acetylenes **10**–**12** in high yields and reaction times of about 30 min while the subsequent TMS group removal using TBAF (tetrabutylammonium fluoride) afforded desired π-linkers **7c**–**9c** (Scheme 2, Table 1, entries 10–12). A similar synthesis of the linear π-linker **9c** has already been reported [40]. Moreover, unprotected terminal acetylenes **7c**–**9c** showed good stability with no signs of decomposition upon standing over several months which facilitate their storage and use.

## Conclusion

It has been shown that extended, donor-substituted π-conjugated linkers can be easily prepared using commercially available precursors under either conventional or modern synthetic conditions. The reaction procedures reported here refer to the optimized procedures for each class of derivatives. Overall 12 extended π-linkers have been easily synthesized (8 of them are new compounds) utilizing procedures such as a lithiation/reaction with triisopropyl borate/esterification with pinacol, Mizoroki–Heck coupling with vinylboronate pinacol ester, borylation with bis(pinacolato)diboron or Sonogashira coupling. Further application of the above boronic esters as well as terminal acetylenes for the construction of imidazole-based D-π-A systems by Suzuki–Miyaura and Sonogashira reaction is currently in progress in our laboratory.

## Supporting Information

File 1 Experimental procedures and characterization of compounds.

File 2 ^1^H NMR spectra as well as GC/MS records for target compounds **3**–**9**.
