# Supplementary material for: Convenient methods for preparing π-conjugated linkers as building blocks for modular chemistry
Source: Beilstein J Org Chem. 2009 Apr 14;5:11. doi: 10.3762/bjoc.5.11 (PMC2686302; doi:10.3762/bjoc.5.11)
Supplement: File 1 — Experimental procedures and characterization of compounds. [file Beilstein_J_Org_Chem-05-11-s001.doc]

**Supporting Information**

# Convenient methods for preparing π‑conjugated linkers as building blocks for modular chemistry

Jiří Kulhánek, Filip Bureš* and Miroslav Ludwig

Address: Institute of Organic Chemistry and Technology, Faculty of Chemical Technology, University of Pardubice, nám. Čs. legií 565, Pardubice, 532 10, Czech Republic

Email: Filip Bureš* - filip.bures@upce.cz

* Corresponding author

**Experimental procedures and characterization of compounds**

Reagents and solvents were reagent-grade and were purchased from Penta, Aldrich, and Acros and used as received. Boronic acids/esters **1a**–**c**, terminal acetylenes **2a**–**c**, styryl dioxaborolanes **3a**–**b**, vinylboronate pinacol ester and pin_2_B_2_ were purchased from Aldrich. Column chromatography was carried out with silica gel 60 (particle size 0.040–0.063 mm, 230–400 mesh; Merck) and commercially available solvents. Thin-layer chromatography (TLC) was conducted on aluminum sheets coated with silica gel 60 F_254_ obtained from Merck, with visualization by UV lamp (254 or 360 nm). Melting points (mp) were measured on a Büchi B-540 melting-point apparatus in open capillaries and are uncorrected. ^1^H and ^13^C NMR spectra were recorded in CDCl_3_ at 360/500 MHz and 90/125 MHz, respectively, with a Bruker AMX 360 or Bruker AVANCE 500 instrument at 25 °C. Chemical shifts are reported in ppm relative to the signal of Me_4_Si. The residual solvent signal in the ^1^H and ^13^C NMR spectra was used as an internal reference (CDCl_3_ – 7.25 and 77.23 ppm). Coupling constants (*J*) are given in Hz. The apparent resonance multiplicity is described as s (singlet), d (doublet), t (triplet), q (quartet) and m (multiplet). In some cases, the carbon atoms with appended boron moiety were not observed in ^13^C NMR spectra. The mass spectra were measured on a GC/MS configuration comprised of an Agilent Technologies – 6890N gas chromatograph (HP-5MS column, length 30 m, I.D. 0.25 mm, film 0.25 µm) equipped with a 5973 Network MS detector (EI 70 eV, mass range 33−550 Da). Elemental analyses were performed on an EA 1108 Fisons instrument.

## Method A

Pd[P(*t*-Bu)_3_]_2_ (38 mg, 0.074 mmol) was added to a solution of vinylboronate pinacol ester (231 mg, 1.5 mmol), 4-bromo-*N*,*N*-dimethylaniline (290 mg, 1.45 mmol) and diisopropylamine (0.84 mL, 6.03 mmol) in dry toluene (8 mL) and the mixture was stirred under Ar at 80 °C for 1 h. After cooling to room temperature, the reaction mixture was quenched with a sat. solution of NH_4_Cl (20 mL, aq), the crude product was extracted with EtOAc (2 × 30 mL), dried (Na_2_SO_4_), concentrated in vacuo and purified by column chromatography (hexane/EtOAc, 4:1).

**(*E*)-2-[4-(*N*,*N*-Dimethylamino)styryl]-4,4,5,5-tetramethyl-1,3,2-dioxaborolane (3c).** White solid. Yield 289 mg (73%); mp 130–133 °C; *R_f_* = 0.40 (silica gel; hexane/EtOAc, 4:1). ^1^H NMR (360 MHz, CDCl_3_, 25 °C): *δ* = 1.29 (s, 12H, CH_3_), 2.97 (s, 6H, CH_3_), 5.90 (d, 1H, *J* = 17.6 Hz, CH), 6.65 (d, 2H, *J* = 8.7 Hz, Ph-H), 7.33 (d, 1H, *J* = 17.6 Hz, CH), 7.38 (d, 2H, *J* = 8.7 Hz, Ph-H). ^13^C NMR (90 MHz, CDCl_3_, 25 °C): *δ* = 25.03, 40.51, 83.22, 112.16, 126.14, 128.61, 150.00, 151.16 (one signal missing). EI-MS (70 eV) *m/z* (rel. int.): 273 (M^+^, 100), 200 (18), 172 (29), 157 (46). Elemental Anal. Calcd for C_16_H_24_BNO_2_ (273.18): C,70.35; H, 8.86; N, 5.13. Found: C, 70.15; H, 8.56; N, 4.98.

## Method B

*n*-BuLi (0.5 mL, 0.8 mmol, 1.6 M solution in hexane) was added dropwise to a solution of substituted 4‑bromobiphenyl (0.8 mmol) in dry THF (20 mL) at −78 °C and the mixture was stirred under Ar for 1 h whereupon triisopropyl borate (0.2 mL; 0.85 mmol) was added and the reaction mixture was stirred at 25 °C for an additional 12 h. The reaction was quenched with diluted HCl (20 mL), the crude boronic acid extracted with EtOAc (2 × 30 mL), concentrated in vacuo and taken up in ether (30 mL). Pinacol (120 mg; 1.02 mmol) was added and the solution was stirred for 12 h at 25 °C, quenched with water, the dioxaborolane was extracted with ether (2 × 30 mL), dried (Na_2_SO_4_) and concentrated in vacuo. The crude product was purified by column chromatography.

**2-(4'-Methoxybiphenyl-4-yl)-4,4,5,5-tetramethyl-1,3,2-dioxaborolane (4b).** The title compound was synthesized from 4-bromo-4’-methoxybiphenyl following the general procedure as a white solid. Yield 201 mg (81%); mp 130−134 °C; *R_f_* = 0.80 (silica gel; hexane/EtOAc, 2:1). ^1^H NMR (500 MHz, CDCl_3_, 25 °C): *δ* = 1.34 (s, 12H, CH_3_), 3.83 (s, 3H, CH_3_), 6.95 (d, 2H, *J* = 6.7 Hz, Ph-H), 7.51−7.56 (m, 4H, Ph-H), 7.84 (d, 2H, *J* = 6.7 Hz, Ph-H). ^13^C NMR (125 MHz, CDCl_3_, 25 °C): *δ* = 25.06, 55.56, 84.00, 114.42, 126.19, 128.45, 133.69, 135.44, 143.68, 159.58 (one signal missing). EI-MS (70 eV) *m/z* (rel. int.): 310 (M^+^, 100), 224 (18), 210 (86), 195 (16), 167 (23). Elemental Anal. Calcd for C_19_H_23_BO_3_ (310.20): C,73.57; H, 7.47. Found: C, 73.65; H, 7.58.

**2-[4'-(*N*,*N*-dimethylamino)biphenyl-4-yl]-4,4,5,5-tetramethyl-1,3,2-dioxaborolane (4c).** The title compound was synthesized from 4-bromo-4’-(*N*,*N*-dimethylamino)biphenyl following the general procedure as a white solid. Yield 214 mg (83%); mp 205−209 °C; *R_f_* = 0.55 (silica gel; hexane/EtOAc, 4:1). ^1^H NMR (360 MHz, CDCl_3_, 25 °C): *δ* = 1.35 (s, 12H, CH_3_), 2.99 (s, 6H, CH_3_), 6.81−6.86 (m, 2H, Ph-H), 7.53−7.57 (m, 4H, Ph-H), 7.83 (d, 2H, *J* = 7.7 Hz, Ph-H). ^13^C NMR (125 MHz, CDCl_3_, 25 °C): *δ* = 25.08, 40.92, 83.91, 113.10, 125.7, 128.07, 128.88, 135.4, 143.97 (two signals missing). EI-MS (70 eV) *m/z* (rel. int.): 323 (M^+^, 100), 265 (16), 222 (21). Elemental Anal. Calcd for C_20_H_26_BNO_2_ (323.24): C,74.32; H, 8.11; N, 4.33. Found: C, 74.24; H, 7.98; N, 4.32.

## Method C

*Preparation of (E)-1-bromo-4-styrylbenzenes.* Synthetic method and full spectral characterization of (*E*)-1-bromo-4-[4-(*N*,*N*-dimethylamino)styryl]benzene were already reported [1]. (*E*)‑1-Bromo-4-(4-methoxystyryl)benzene was synthesized analogously with only prolonged isomerisation for up to 48 h.

**(*E*)‑1-Bromo-4-(4-methoxystyryl)benzene.** White solid, yield 37%; mp 191−194 °C; *R_f_* = 0.50 (silica gel; hexane/EtOAc, 4:1). ^1^H NMR (500 MHz, CDCl_3_, 25 °C): *δ* = 3.80 (s, 3H, CH_3_), 6.85−6.89 (m, 3H, CH+Ph-H), 7.02 (d, 1H, *J* = 16.3 Hz, CH), 7.32 (d, 2H, *J* = 8.6 Hz, Ph-H), 7.41−7.44 (m, 4H, Ph-H). ^13^C NMR (125 MHz, CDCl_3_, 25 °C): *δ* = 55.52, 114.36, 120.96, 125.46, 127.89, 127.98, 129.10, 129.93, 131.89, 136.78, 159.65. EI-MS (70 eV) *m/z* (rel. int.): 288 (M^+^, 100), 165 (95). Elemental Anal. Calcd for C_15_H_31_BrO (289.17): C,62.30; H, 4.53; Br, 27.63. Found: C, 62.45; H, 4.60; Br, 27.69.

*Reaction with pin_2_B_2_.* PdCl_2_(dppf) (23 mg, 0.031 mmol) was added to a solution of bis(pinacolato)diboron (298 mg, 1.18 mmol), 4-substituted (*E*)-1-bromo-4-styrylbenzene (1.1 mmol), and potassium acetate (314 mg, 3.2 mmol) in DMSO (6 mL)/dioxane (2 mL) and the mixture was stirred under Ar at 80 °C for 4 h. After cooling to room temperature, the reaction mixture was quenched with a sat. solution of NH_4_Cl (20 mL, aq), the crude product was extracted with EtOAc (2 × 30 mL), dried (Na_2_SO_4_), concentrated in vacuo and purified by column chromatography.

**(*E*)-2-(4-(4-Methoxystyryl)phenyl)-4,4,5,5-tetramethyl-1,3,2-dioxaborolane (5b).** The title compound was synthesized from (*E*)‑1-bromo-4-(4-methoxystyryl)benzene following the general procedure as a white solid. Yield 255 mg (69%); mp 165−169 °C; *R_f_* = 0.70 (silica gel; hexane/EtOAc, 4:1). ^1^H NMR (360 MHz, CDCl_3_, 25 °C): *δ* = 1.34 (s, 12H, CH_3_), 3.82 (s, 3H, CH_3_), 6.89 (d, 2H, *J* = 8.0 Hz, Ph-H), 6.97 (d, 1H, *J* = 16.5 Hz, CH), 7.13 (d, 1H, *J* = 16.5 Hz, CH), 7.44−7.49 (m, 4H, Ph-H), 7.77 (d, 2H, *J* = 7.0 Hz, Ph-H). ^13^C NMR (90 MHz, CDCl_3_, 25 °C): *δ* = 25.09, 55.55, 83.96, 114.38, 125.75, 126.74, 128.07, 129.40, 131.81, 135.35, 140.59, 159.66 (one signal missing). EI-MS (70 eV) *m/z* (rel. int.): 336 (M^+^, 100), 236 (22), 207 (19), 165 (16). Elemental Anal. Calcd for C_21_H_25_BO_3_ (336.19): C,75.01; H, 7.49. Found: C, 75.18; H, 7.58.

**(*E*)-2-{4-[4-(*N*,*N*-Dimethylamino)styryl]phenyl}-4,4,5,5-tetramethyl-1,3,2-dioxaborolane (5c).** The title compound was synthesized from (*E*)-1-bromo-4-[4-(*N*,*N*-dimethylamino)styryl]benzene following the general procedure as a white solid. Yield 315 mg (82%); mp 181−183 °C; *R_f_* = 0.80 (silica gel; hexane/EtOAc, 2:1). ^1^H NMR (360 MHz, CDCl_3_, 25 °C): *δ* = 1.34 (s, 12H, CH_3_), 2.98 (s, 6H, CH_3_), 6.71 (d, 2H, *J* = 7.6 Hz, Ph-H), 6.91 (d, 1H, *J* = 16.5 Hz, CH), 7.11 (d, 1H, *J* = 16.5 Hz, CH), 7.41 (d, 2H, *J* = 7.9 Hz, Ph-H), 7.46 (d, 2H, *J* = 7.6 Hz, Ph-H), 7.76 (d, 2H, *J* = 7.8 Hz, Ph-H). ^13^C NMR (90 MHz, CDCl_3_, 25 °C): *δ* = 25.09, 40.70, 83.88, 112.66, 124.50, 125.50, 127.95, 130.01, 135.31, 141.17, 151.51 (two signals missing). EI-MS (70 eV) *m/z* (rel. int.): 349 (M^+^, 100), 248 (20). Elemental Anal. Calcd for C_22_H_28_BNO_2_ (349.27): C,75.65; H, 8.08, N, 4.01. Found: C, 75.68; H, 8.10, N, 3.99.

## Method D

PdCl_2_(PPh_3_)_2_ (32 mg, 0.046 mmol) and CuI (17 mg, 0.089 mmol) were added to a solution of 2-(4-iodophenyl)-4,4,5,5-tetramethyl-1,3,2-dioxaborolane (500 mg, 1.5 mmol), **2a**−**c** (1.5 mmol), and TEA (1.1 mL, 7.9 mmol) in THF (15 mL) and the mixture was stirred under Ar at 40 °C for 3 h. After cooling to room temperature, the reaction mixture was quenched with water (20 mL), the crude product was extracted with EtOAc (2 × 30 mL), dried (Na_2_SO_4_), concentrated in vacuo and purified by column chromatography.

**4,4,5,5-Tetramethyl-2-[4-(phenylethynyl)phenyl)]-1,3,2-dioxaborolane (6a).** The title compound was synthesized from **2a** following the general procedure as a white solid. Yield 356 mg (78%); mp 93−95 °C; *R_f_* = 0.80 (silica gel; hexane/EtOAc, 4:1). ^1^H NMR (360 MHz, CDCl_3_, 25 °C): *δ* = 1.35 (s, 12H, CH_3_), 7.32−7.35 (m, 3H, Ph-H), 7.52 (d, 4H, *J* = 7.5 Hz, Ph-H), 7.78 (d, 2H, *J* = 7.7 Hz, Ph-H). ^13^C NMR (90 MHz, CDCl_3_, 25 °C): *δ* = 25.09, 84.17, 89.76, 90.91, 123.38, 126.17, 128.56, 130.98, 131.87, 134.81, 136.49, 137.13. EI-MS (70 eV) *m/z* (rel. int.): 304 (M^+^, 81), 289 (19), 218 (31), 204 (100), 176 (15). Elemental Anal. Calcd for C_20_H_21_BO_2_ (304.19): C,78.97; H, 6.96. Found: C, 78.91; H, 6.98.

**2-{4-[(4-Methoxyphenyl)ethynyl]phenyl}-4,4,5,5-tetramethyl-1,3,2-dioxaborolane (6b).** The title compound was synthesized from **2b** following the general procedure as a white solid. Yield 361 mg (72%); mp 132−134 °C; *R_f_* = 0.70 (silica gel; hexane/EtOAc, 4:1). ^1^H NMR (360 MHz, CDCl_3_, 25 °C): *δ* = 1.34 (s, 12H, CH_3_), 3.82 (s, 3H, CH_3_), 6.87 (d, 2H, *J* = 8.5 Hz, Ph‑H), 7.45−7.51 (m, 4H, Ph-H), 7.76 (d, 2H, *J* = 7.6 Hz, Ph-H). ^13^C NMR (90 MHz, CDCl_3_, 25 °C): *δ* = 25.10, 55.52, 84.14, 88.53, 91.00, 102.24, 114.23, 115.49, 126.53, 130.81, 133.35, 134.79, 159.94. EI-MS (70 eV) *m/z* (rel. int.): 334 (M^+^, 100), 234 (51), 219 (22), 191 (24). Elemental Anal. Calcd for C_21_H_23_BO_3_ (334.22): C,75.47; H, 6.94. Found: C, 75.69; H, 6.83.

**2-(4-{[4-(*N*,*N*-Dimethylamino)phenyl]ethynyl}phenyl)-4,4,5,5-tetramethyl-1,3,2-dioxaborolane (6c).** The title compound was synthesized from **2c** following the general procedure as a white solid. Yield 473 mg (91%); mp 177−179 °C; *R_f_* = 0.45 (silica gel; hexane/EtOAc, 4:1). ^1^H NMR (360 MHz, CDCl_3_, 25 °C): *δ* = 1.34 (s, 12H, CH_3_), 2.98 (s, 6H, CH_3_), 6.64 (d, 2H, *J* = 8.6 Hz, Ph‑H), 7.40 (d, 2H, *J* = 8.6 Hz, Ph-H), 7.48 (d, 2H, *J* = 7.6 Hz, Ph-H), 7.75 (d, 2H, *J* = 7.6 Hz, Ph-H). ^13^C NMR (90 MHz, CDCl_3_, 25 °C): *δ* = 25.10, 40.40, 84.07, 87.88, 92.40, 102.22, 110.08, 112.01, 127.15, 130.62, 133.02, 134.74, 150.4. EI-MS (70 eV) *m/z* (rel. int.): 347 (M^+^, 100), 289 (18), 246 (23). Elemental Anal. Calcd for C_22_H_26_BNO_2_ (347.26): C,76.09; H, 7.55; N, 4.03. Found: C, 75.99; H, 7.51; N, 4.06.

## Method E

PdCl_2_(PPh_3_)_2_ (21 mg, 0.030 mmol), CuI (11 mg, 0.060 mmol), and trimethylsilylacetylene (0.14 mL, 2.0 mmol) were added to a solution of iodo substrate (1.5 mmol) in TEA (3 mL, 21.6 mmol) and THF (20 mL) and the mixture was stirred under Ar at 25 °C for 30 min. The solvent was evaporated in vacuo, the residue taken up in DCM (15 mL) and filtered through a plug (silica gel; DCM) to afford pure TMS-protected acetylenes **10**−**12**.

The above TMS-protected acetylene (1.0 mmol) was dissolved in THF (20 mL), TBAF (1.0 mL; 1 M solution in THF) was added and the reaction mixture stirred for 30 min. The solvent was evaporated in vacuo, the residue taken up in DCM (15 mL) and filtered through a plug (silica gel; DCM) to afford pure products **7c**−**9c**.

***N*,*N*-Dimethyl-4'-[(trimethylsilyl)ethynyl]biphenyl-4-amine (10).** Off-white solid. Yield 431 mg (98%); mp 193−194 °C; *R_f_* = 0.55 (silica gel; hexane/DCM, 1:1). ^1^H NMR (500 MHz, CDCl_3_, 25 °C): *δ* = 0.25 (s, 9H, CH_3_), 2.99 (s, 6H, CH_3_), 6.78 (d, 2H, *J* = 8.8 Hz, Ph‑H), 7.45−7.50 (m, 4H, Ph-H). ^13^C NMR (125 MHz, CDCl_3_, 25 °C): *δ* = 0.27, 40.73, 94.81, 105.65, 112.89, 120.62, 125.98, 127.80, 128.11, 132.54, 141.39, 150.65. EI-MS (70 eV) *m/z* (rel. int.): 293 (M^+^, 100), 278 (65), 262 (22), 139 (31).

**4'-Ethynyl-*N*,*N*-dimethylbiphenyl-4-amine (7c).** Off-white solid. Yield 203 mg (92%); mp 156−157 °C; *R_f_* = 0.60 (silica gel; hexane/DCM, 1:1). ^1^H NMR (500 MHz, CDCl_3_, 25 °C): *δ* = 3.00 (s, 6H, CH_3_), 3.10 (s, 1H, CH), 6.79 (d, 2H, *J* = 8.8 Hz, Ph‑H), 7.49−7.52 (m, 6H, Ph-H). ^13^C NMR (125 MHz, CDCl_3_, 25 °C): *δ* = 40.70, 77.38, 84.16, 112.88, 126.11, 127.83, 127.91, 128.85, 132.69, 141.78, 150.41. EI-MS (70 eV) *m/z* (rel. int.): 221 (M^+^, 100), 205 (15), 176 (19). Elemental Anal. Calcd for C_16_H_15_N (221.30): C,86.84; H, 6.83; N, 6.33. Found: C, 86.95; H, 6.91; N, 6.36.

**(*E*)-*N*,*N*-Dimethyl-4-{4-[(trimethylsilyl)ethynyl]styryl}aniline (11).** Yellowish solid. Yield 464 mg (97%); mp 193−194 °C; *R_f_* = 0.45 (silica gel; hexane/DCM, 1:1). ^1^H NMR (500 MHz, CDCl_3_, 25 °C): *δ* = 0.25 (s, 9H, CH_3_), 2.98 (s, 6H, CH_3_), 6.70 (d, 2H, *J* = 8.9 Hz, Ph‑H), 6.87 (d, 1H, *J* = 16.3 Hz, CH), 7.05 (d, 1H, *J* = 16.3 Hz, CH), 7.38−7.49 (m, 6H, Ph-H). ^13^C NMR (125 MHz, CDCl_3_, 25 °C): *δ* = 0.25, 40.67, 94.66, 105.74, 112.55, 121.09, 123.68, 125.54, 125.91, 127.95, 130.06, 132.42, 138.63, 150.46. EI-MS (70 eV) *m/z* (rel. int.): 319 (M^+^, 100), 304 (26), 152 (27).

**(*E*)-4-(4-Ethynylstyryl)-*N*,*N*-dimethylaniline (8c).** Yellowish solid. Yield 225 mg (91%); mp 189−190 °C; *R_f_* = 0.45 (silica gel; hexane/DCM, 1:1). ^1^H NMR (500 MHz, CDCl_3_, 25 °C): *δ* = 2.99 (s, 6H, CH_3_), 3.11 (s, 1H, CH), 6.71 (d, 2H, *J* = 8.9 Hz, Ph‑H), 6.87 (d, 1H, *J* = 16.3 Hz, CH), 7.06 (d, 1H, *J* = 16.3 Hz, CH), 7.39−7.48 (m, 6H, Ph-H). ^13^C NMR (125 MHz, CDCl_3_, 25 °C): *δ* = 40.68, 77.65, 84.24, 112.55, 120.04, 123.55, 125.46, 125.99, 127.98, 130.28, 132.58, 138.98, 150.50. EI-MS (70 eV) *m/z* (rel. int.): 247 (M^+^, 100), 231 (12), 202 (20). Elemental Anal. Calcd for C_18_H_17_N (247.33): C,87.41; H, 6.93; N, 5.66. Found: C, 87.55; H, 6.95; N, 5.56.

***N*,*N*-Dimethyl-4-{[4-[(trimethylsilyl)ethynyl]phenyl]ethynyl}aniline (12).** Off-white solid. Yield 471 mg (99%); mp 129−130 °C; *R_f_* = 0.30 (silica gel; hexane/DCM, 4:1). ^1^H NMR (500 MHz, CDCl_3_, 25 °C): *δ* = 0.25 (s, 9H, CH_3_), 2.99 (s, 6H, CH_3_), 6.64 (d, 2H, *J* = 9.0 Hz, Ph‑H), 7.39−7.41 (m, 6H, Ph-H). ^13^C NMR (125 MHz, CDCl_3_, 25 °C): *δ* = 0.16, 40.38, 87.40, 93.04, 95.89, 105.14, 109.82, 111.98, 122.08, 124.54, 131.19, 132.02, 132.98, 150.40. EI-MS (70 eV) *m/z* (rel. int.): 317 (M^+^, 100), 302 (36), 286 (20), 151 (28).

**4-[(4-Ethynylphenyl)ethynyl]-*N*,*N*-dimethylaniline (9c).** Off-white solid. Yield 218 (89%); mp 163−164 °C (Lit. [2] mp 138−140 °C); *R_f_* = 0.20 (silica gel; hexane/DCM, 4:1). ^1^H and ^13^C NMR spectra were consistent with those reported in the literature [2]. EI-MS (70 eV) *m/z* (rel. int.): 245 (M^+^, 100), 229 (19), 200 (19).

## General procedure for the conversion of ArBr to ArI derivatives

*n*-BuLi (0.66 mL, 1.05 mmol, 1.6 M solution in hexane) was added dropwise to a solution of substituted 4-bromobiphenyl or 4-bromostilbene (1.0 mmol) in dry THF (20 mL) at −78 °C and the mixture was stirred under Ar for 30 min whereupon iodine (533 mg; 2.1 mmol) was added and the reaction mixture was stirred at 25 °C for an additional 2 h. The solvent was evaporated, the residue taken up in DCM, washed with sat. NaHSO_3_ (aq), dried (Na_2_SO_4_) and concentrated in vacuo to afford iodo‑derivative which was used directly in the next step without further purification.

# References

1. Oehlke, A.; Auer, A. A.; Jahre, I.; Walfort, B.; Rüffer, T.; Zoufalá, P.; Lang, H.; Spange, S. *J. Org. Chem.* **2007,** *72,* 4328–4339. doi:[10.1021/jo070084v](http://dx.doi.org/10.1021/jo070084v)
2. Liu, Z.-q.; Fang, Q.; Wang, D.; Cao, D.-x.; Xue, G.; Yu, W.-t.; Lei, H. *Chem.–Eur. J.* **2003,** *9,* 5074–5084. doi:[10.1002/chem.200304833](http://dx.doi.org/10.1002/chem.200304833)
