# Supplementary material for: Convenient methods for preparing π-conjugated linkers as building blocks for modular chemistry
Source: Beilstein J Org Chem. 2009 Apr 14;5:11. doi: 10.3762/bjoc.5.11 (PMC2686302; doi:10.3762/bjoc.5.11)
Supplement: File 2 — 1H NMR spectra as well as GC/MS records for target compounds 3–9. [file Beilstein_J_Org_Chem-05-11-s002.pdf]

Supporting Information

Convenient methods for preparing π-conjugated linkers as building blocks for modular chemistry

Jiří Kulhánek, Filip Bureš* and Miroslav Ludwig

Address: Institute of Organic Chemistry and Technology, Faculty of Chemical Technology, University of Pardubice, nám. Čs. legií 565, Pardubice, 532 10, Czech Republic

Email: Filip Bureš* - filip.bures@upce.cz

* Corresponding author

Table of Contents **Page**

1. ^{1}H NMR spectrum and GC/MS record for **3c** S2
2. ^{1}H NMR spectrum and GC/MS record for **4b** S3
3. ^{1}H NMR spectrum and GC/MS record for **4c** S4
4. ^{1}H NMR spectrum and GC/MS record for **5b** S5
5. ^{1}H NMR spectrum and GC/MS record for **5c** S6
6. ^{1}H NMR spectrum and GC/MS record for **6a** S7
7. ^{1}H NMR spectrum and GC/MS record for **6b** S8
8. ^{1}H NMR spectrum and GC/MS record for **6c** S9
9. ^{1}H NMR spectrum and GC/MS record for **7c** S10
10. ^{1}H NMR spectrum and GC/MS record for **8c** S11
11. ^{1}H NMR spectrum and GC/MS record for **9c** S12

1. ^{1}H NMR spectrum (360 MHz, $CDCl_3$, 25 °C) and GC/MS record for **3c**

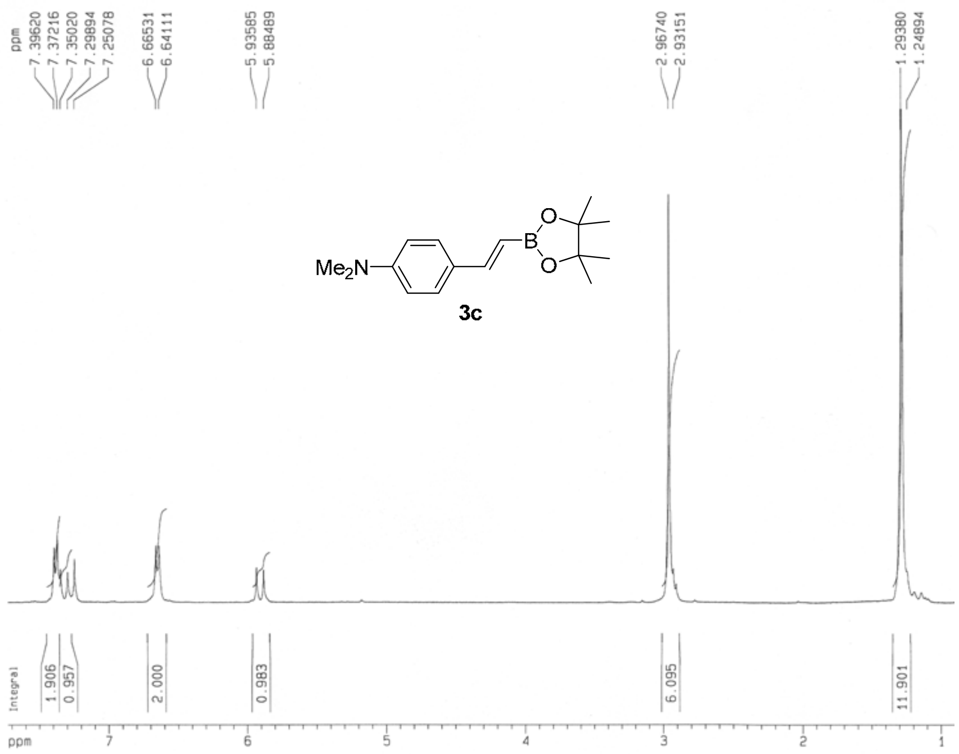

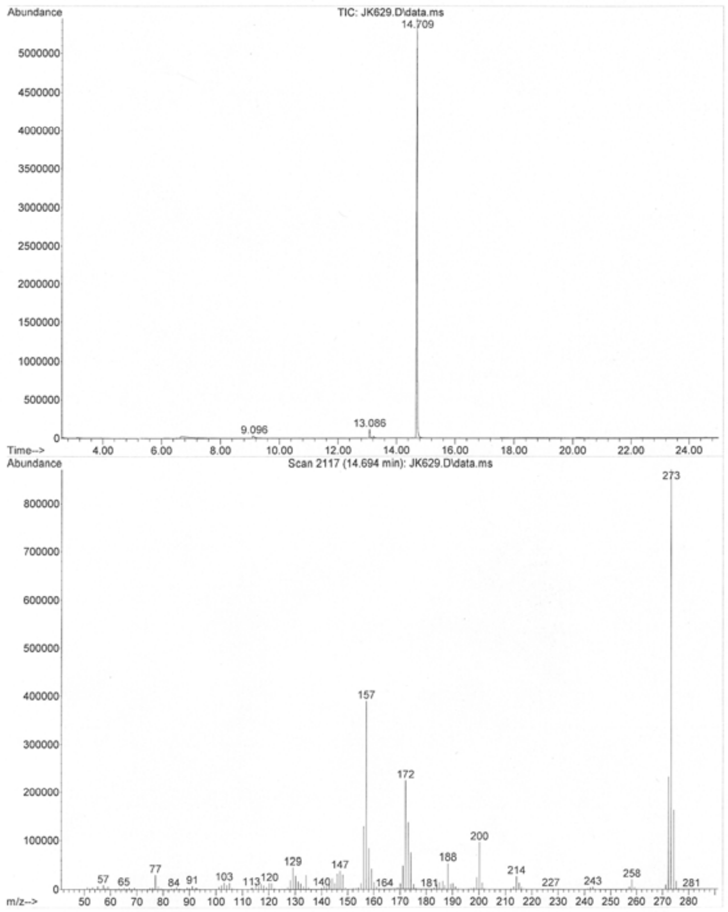

2. ^{1}H NMR spectrum (500 MHz, $CDCl_3$, 25 °C) and GC/MS record for **4b**

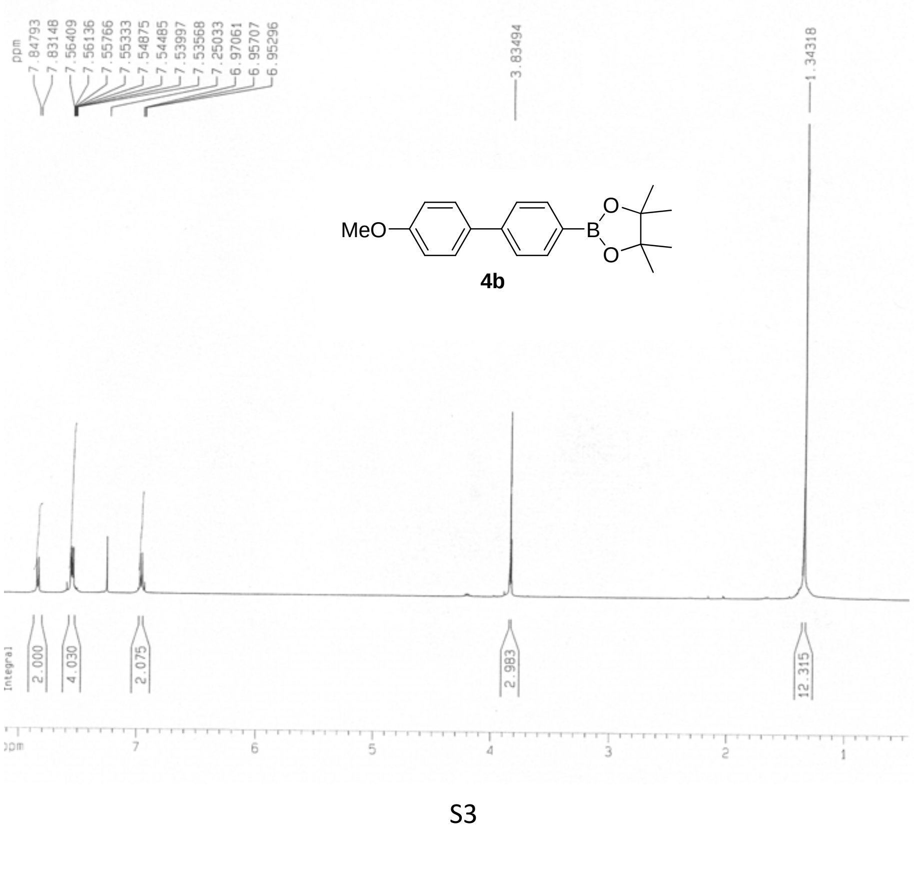

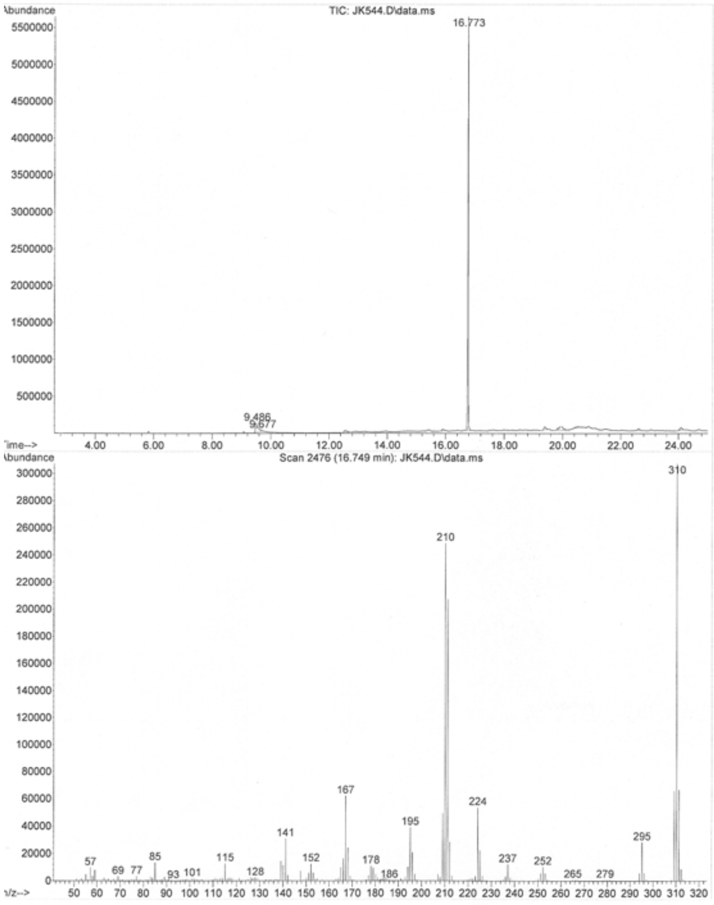

3. ^{1}H NMR spectrum (360 MHz, $CDCl_3$, 25 °C) and GC/MS record for **4c**

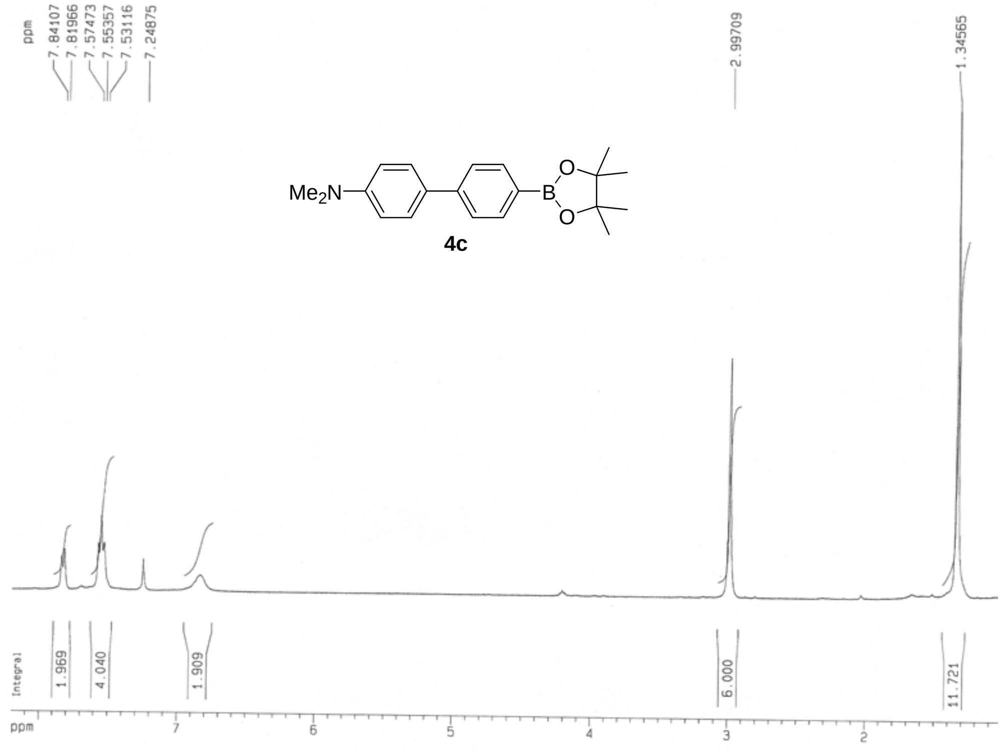

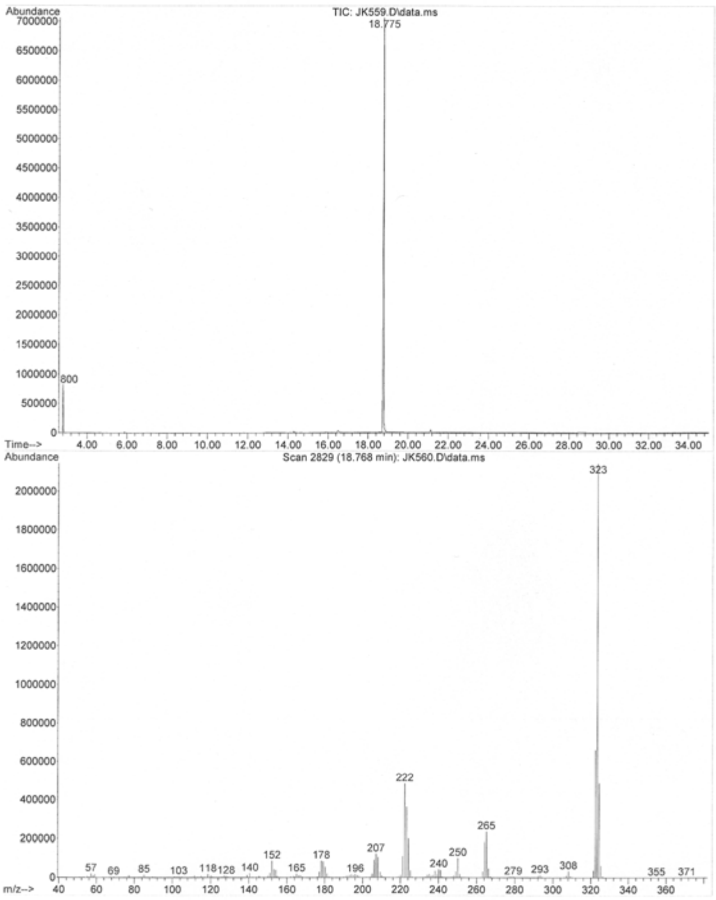

4. ^{1}H NMR spectrum (360 MHz, $CDCl_3$, 25 °C) and GC/MS record for **5b**

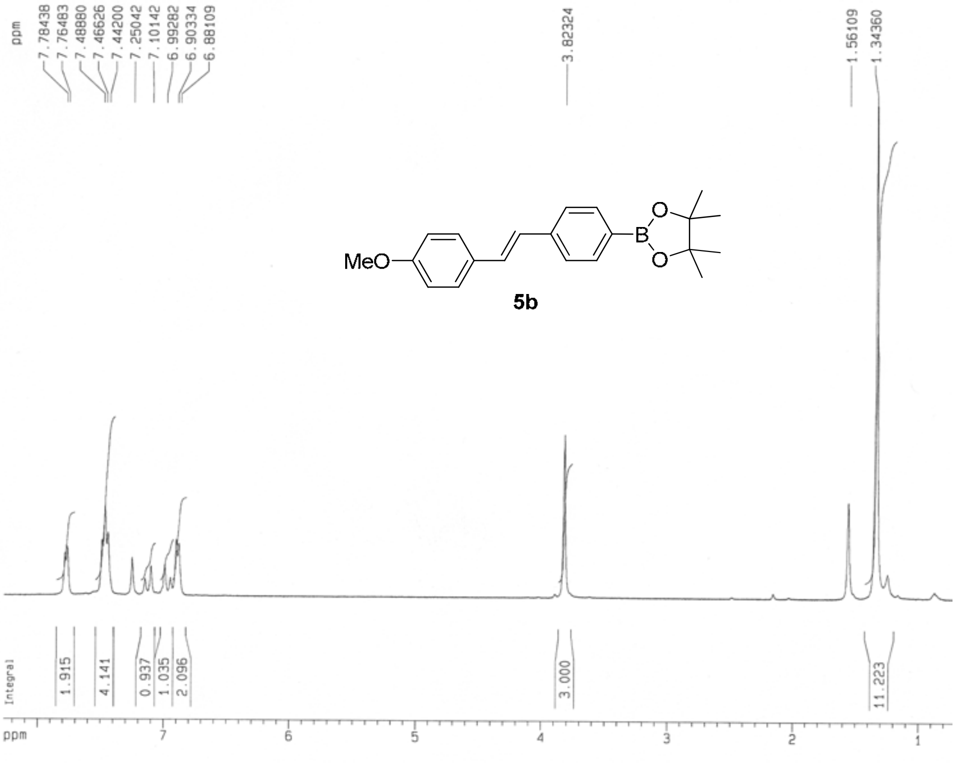

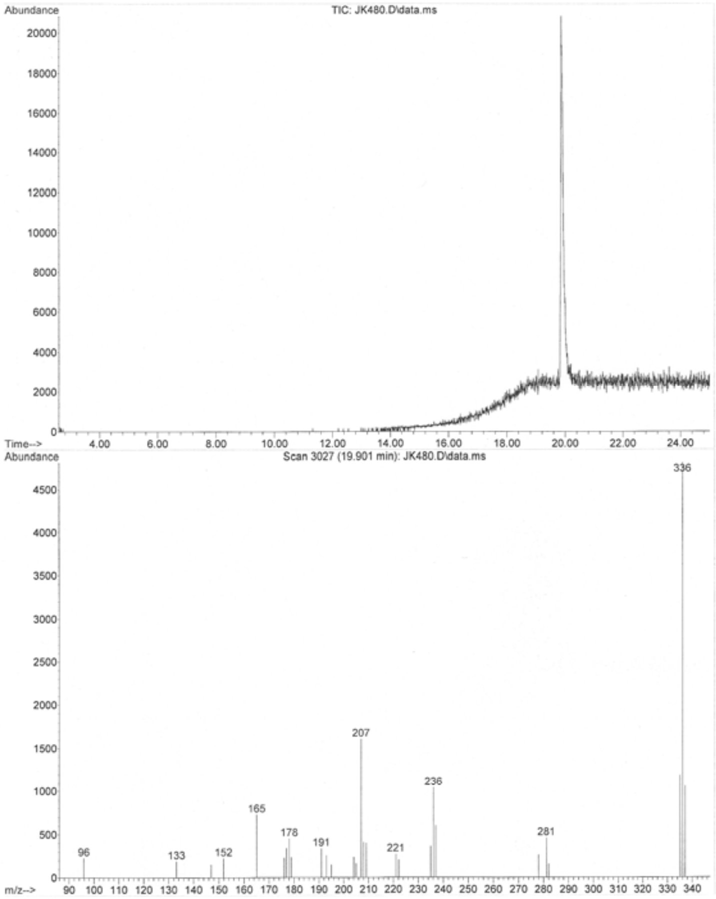

5. ^{1}H NMR spectrum (360 MHz, $CDCl_3$, 25 °C) and GC/MS record for **5c**

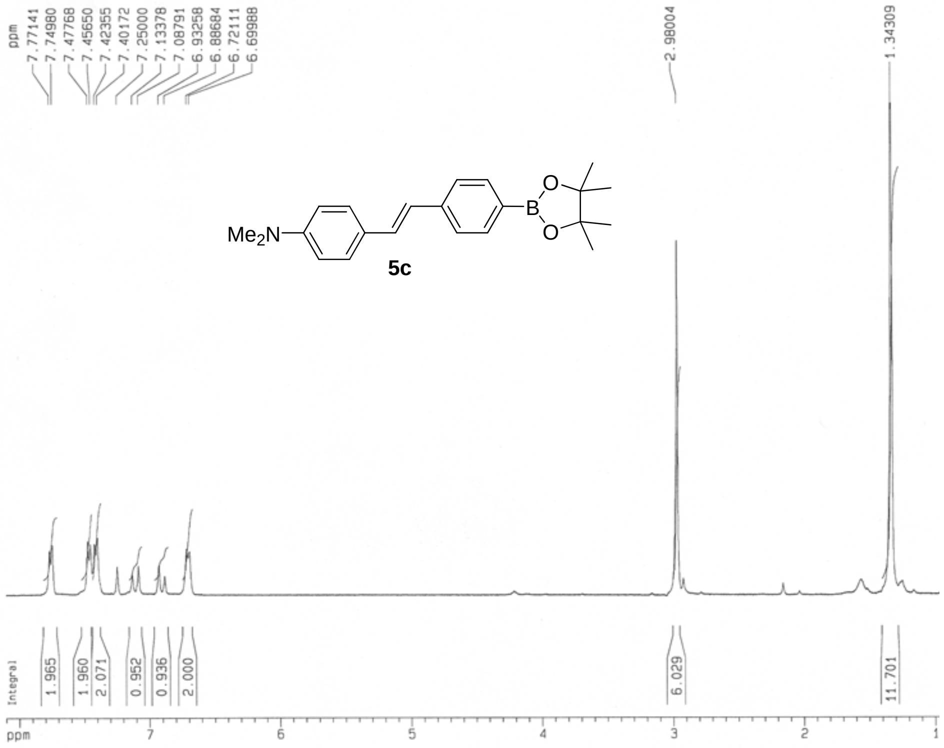

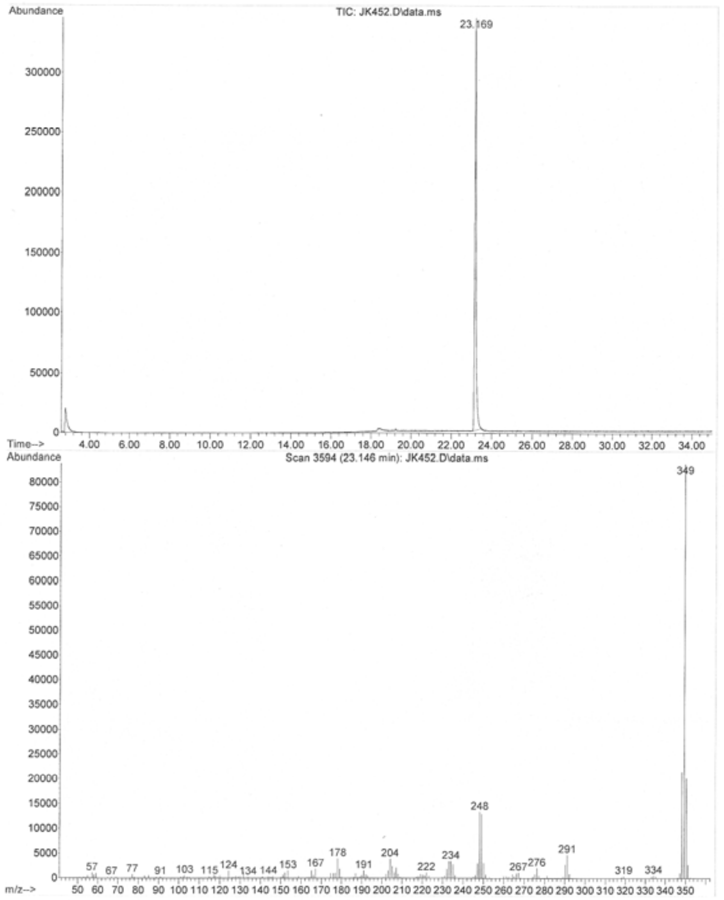

6. ^{1}H NMR spectrum (360 MHz, $CDCl_3$, 25 °C) and GC/MS record for **6a**

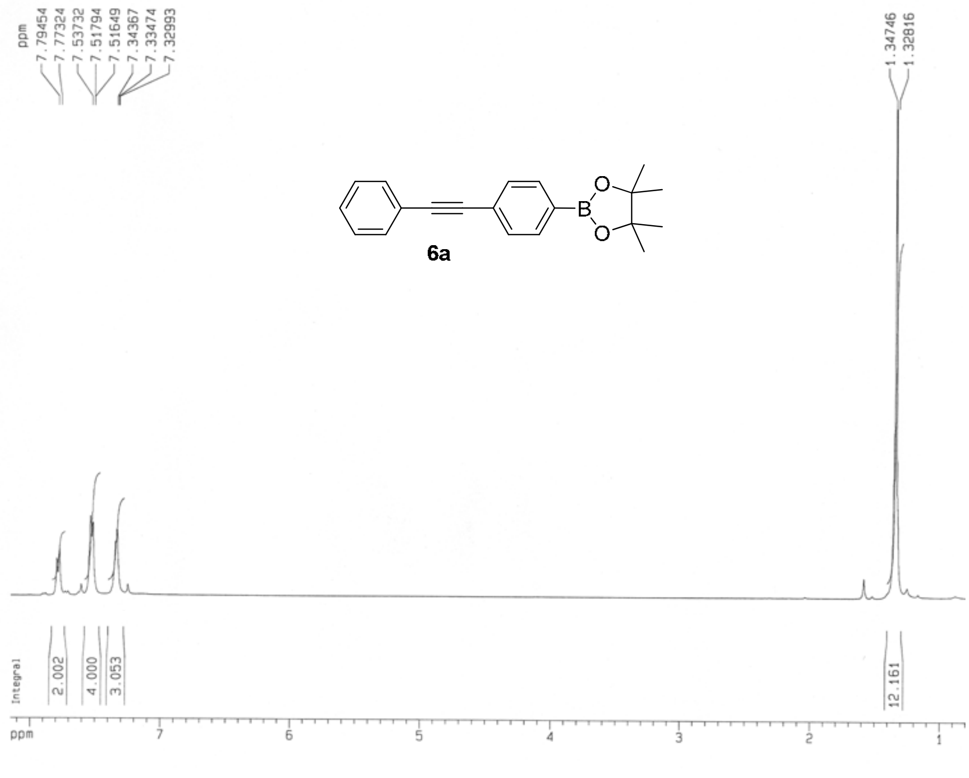

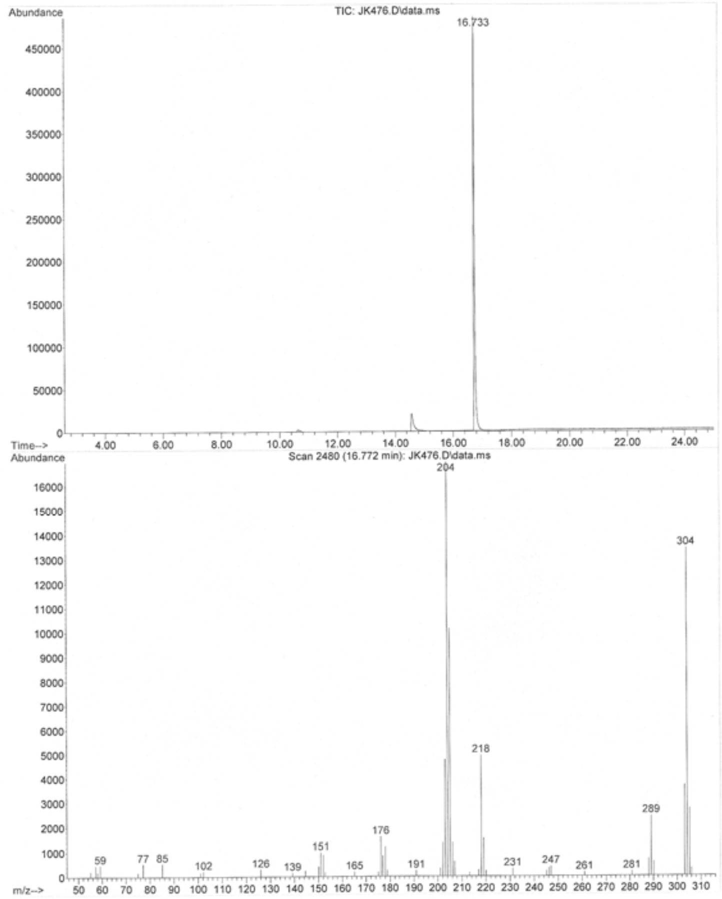

7. ^{1}H NMR spectrum (360 MHz, $CDCl_3$, 25 °C) and GC/MS record for **6b**

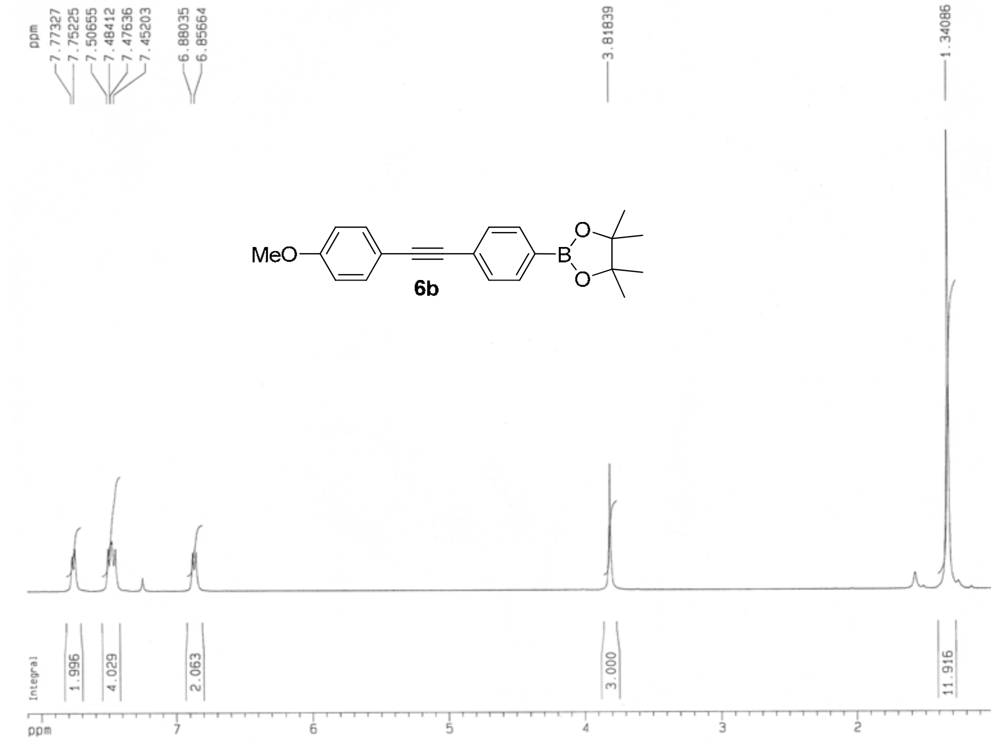

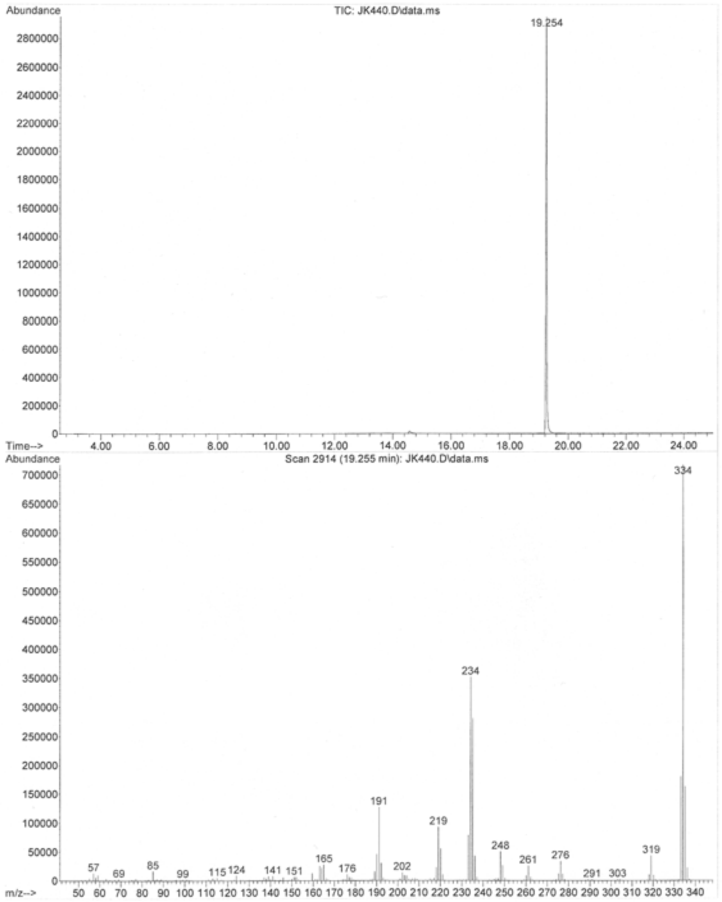

8. ^{1}H NMR spectrum (360 MHz, $CDCl_3$, 25 °C) and GC/MS record for **6c**

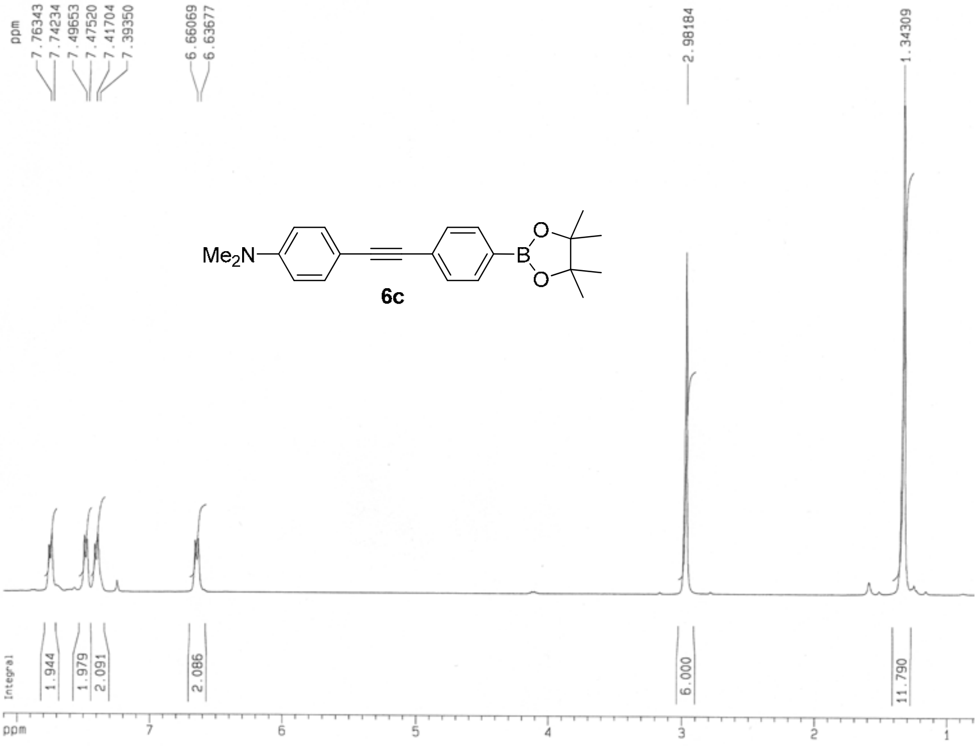

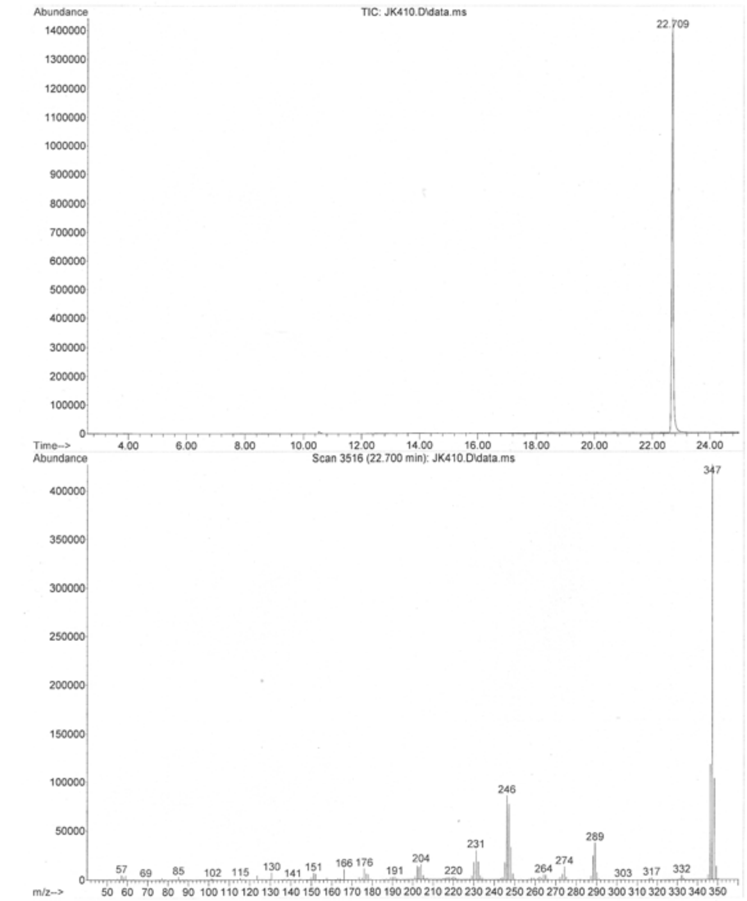

9. ^{1}H NMR spectrum (360 MHz, $CDCl_3$, 25 °C) and GC/MS record for **7c**

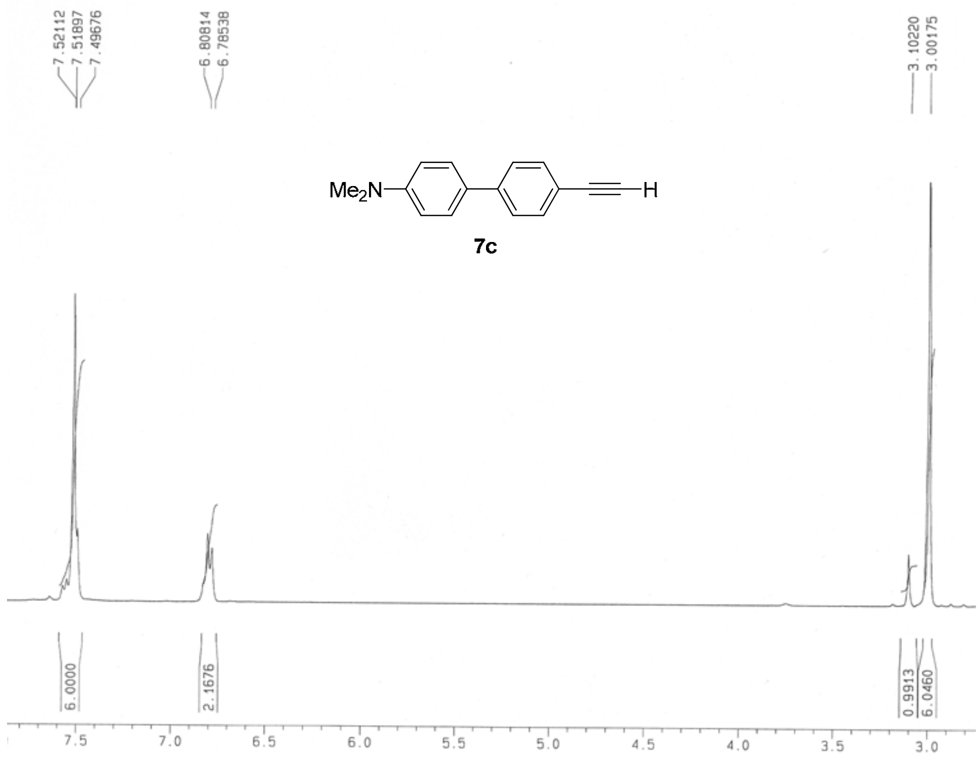

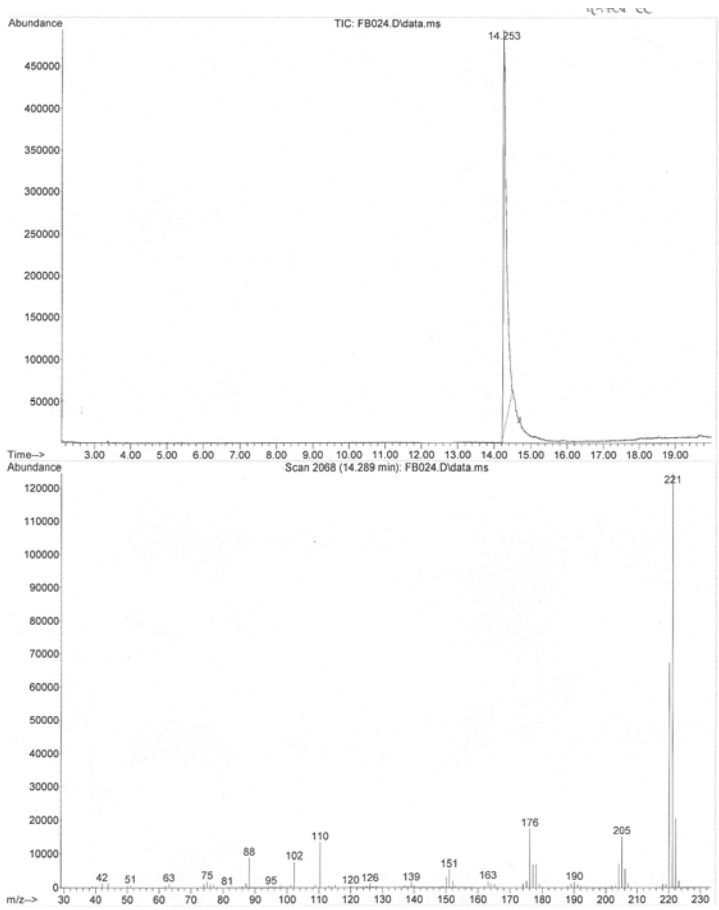

10. ^{1}H NMR spectrum (500 MHz, $CDCl_3$, 25 °C) and GC/MS record for **8c**

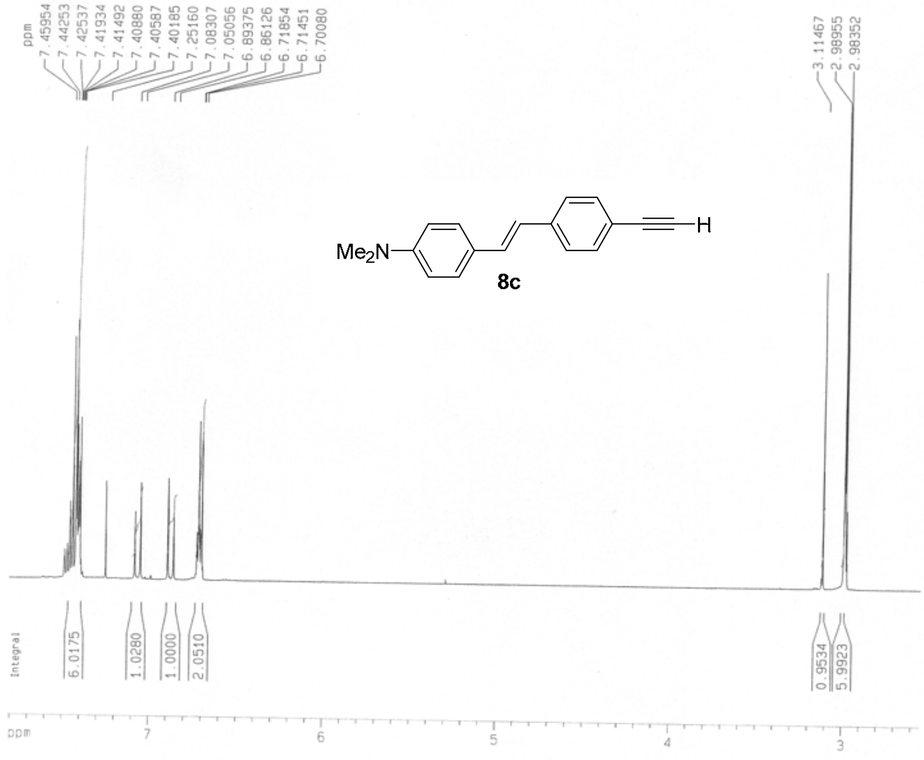

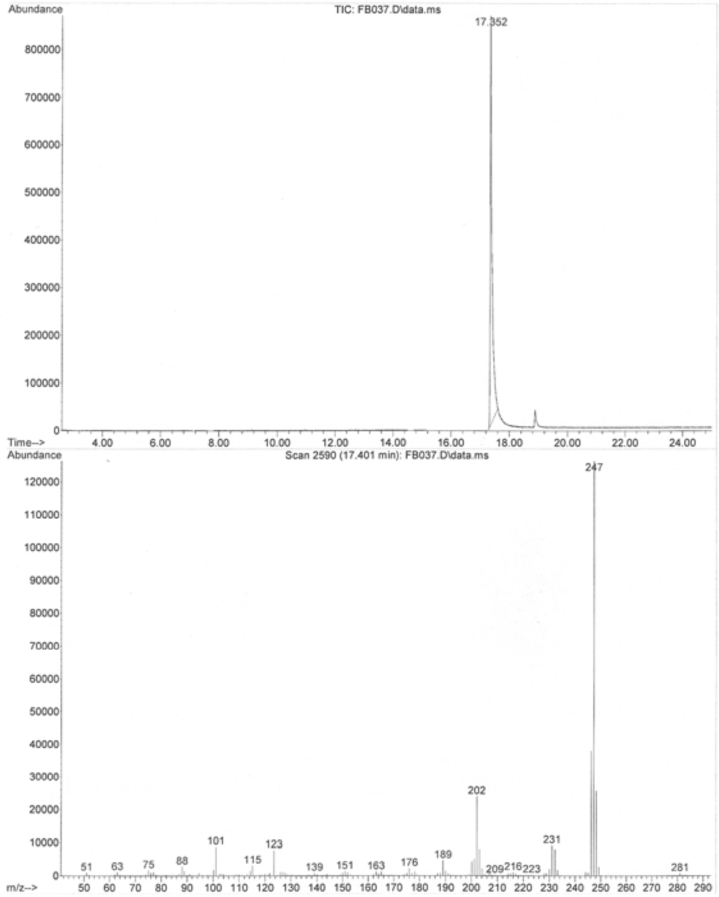

11. ^{1}H NMR spectrum (500 MHz, $CDCl_3$, 25 °C) and GC/MS record for **9c**

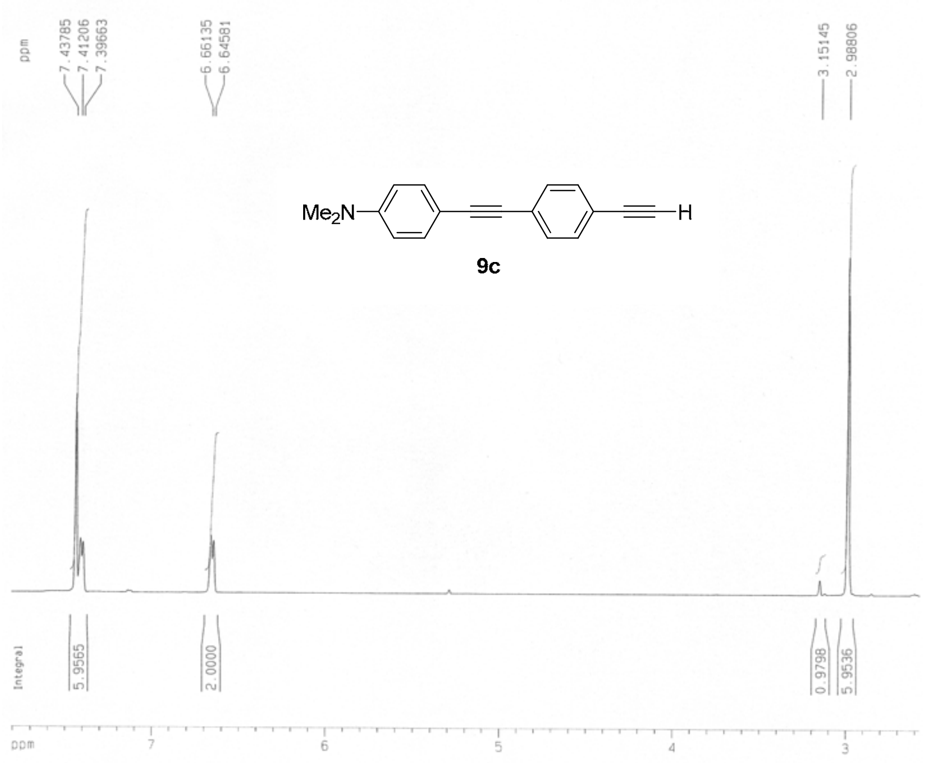

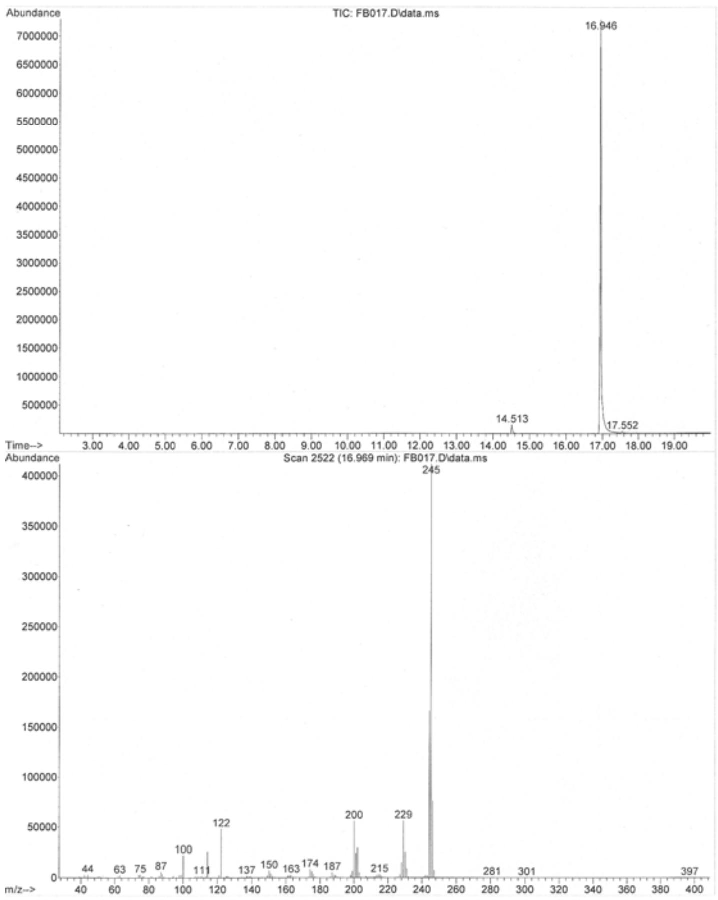

Abundance
TIC: FB017.D\data.ms
16.946
14.513
17.552
Time-->
Abundance
Scan 2522 (16.969 min): FB017.D\data.ms
245
200
229
122
100
44
63
75
87
111
137
150
163
174
187
215
281
301
397
m/z-->
